# *Geobacter* Strains Expressing Poorly Conductive Pili Reveal Constraints on Direct Interspecies Electron Transfer Mechanisms

**DOI:** 10.1128/mBio.01273-18

**Published:** 2018-07-10

**Authors:** Toshiyuki Ueki, Kelly P. Nevin, Amelia-Elena Rotaru, Li-Ying Wang, Joy E. Ward, Trevor L. Woodard, Derek R. Lovley

**Affiliations:** a Department of Microbiology, University of Massachusetts, Amherst, Massachusetts, USA; b Department of Biology, University of Southern Denmark, Odense, Denmark; University of Delaware

**Keywords:** DIET, coculture, extracellular electron transfer, syntrophy

## Abstract

Cytochrome-to-cytochrome electron transfer and electron transfer along conduits of multiple extracellular magnetite grains are often proposed as strategies for direct interspecies electron transfer (DIET) that do not require electrically conductive pili (e-pili). However, physical evidence for these proposed DIET mechanisms has been lacking. To investigate these possibilities further, we constructed *Geobacter metallireducens* strain Aro-5, in which the wild-type pilin gene was replaced with the *aro-5* pilin gene that was previously shown to yield poorly conductive pili in *Geobacter sulfurreducens* strain Aro-5. *G. metallireducens* strain Aro-5 did not reduce Fe(III) oxide and produced only low current densities, phenotypes consistent with expression of poorly conductive pili. Like *G. sulfurreducens* strain Aro-5, *G. metallireducens* strain Aro-5 displayed abundant outer surface cytochromes. Cocultures initiated with wild-type G. metallireducens as the electron-donating strain and *G. sulfurreducens* strain Aro-5 as the electron-accepting strain grew via DIET. However, G. metallireducens Aro-5/G. sulfurreducens wild-type cocultures did not. Cocultures initiated with the Aro-5 strains of both species grew only when amended with granular activated carbon (GAC), a conductive material known to be a conduit for DIET. Magnetite could not substitute for GAC. The inability of the two Aro-5 strains to adapt for DIET in the absence of GAC suggests that there are physical constraints on establishing DIET solely through cytochrome-to-cytochrome electron transfer or along chains of magnetite. The finding that DIET is possible with electron-accepting partners that lack highly conductive pili greatly expands the range of potential electron-accepting partners that might participate in DIET.

## IMPORTANCE

DIET is thought to be an important mechanism for interspecies electron exchange in natural anaerobic soils and sediments in which methane is either produced or consumed, as well as in some photosynthetic mats and anaerobic digesters converting organic wastes to methane. Understanding the potential mechanisms for DIET will not only aid in modeling carbon and electron flow in these geochemically significant environments but will also be helpful for interpreting meta-omic data from as-yet-uncultured microbes in DIET-based communities and for designing strategies to promote DIET in anaerobic digesters. The results demonstrate the need to develop a better understanding of the diversity of types of e-pili in the microbial world to identify potential electron-donating partners for DIET. Novel methods for recovering as-yet-uncultivated microorganisms capable of DIET in culture will be needed to further evaluate whether DIET is possible without e-pili in the absence of conductive materials such as GAC.

## INTRODUCTION

Direct interspecies electron transfer (DIET) appears to be an important form of syntrophy in diverse anaerobic environments (1–5). These include anaerobic digesters converting organic waste to methane (6, 7), methanogenic soils/sediments (8–10), and possibly photosynthetic mats (11) and marine sediments in which methane is oxidized with the reduction of sulfate (12–15). Thus, a better understanding of the mechanisms for DIET would provide new insights into the functioning of anaerobic environments of practical and geochemical significance.

**FIG 1:**
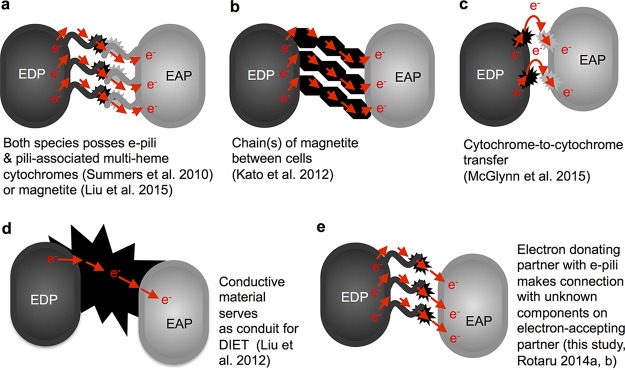
Models for direct interspecies electron transfer (DIET) between electron-donating (EDP) and electron-accepting (EAP) partners. Previously proposed models (a to d) as well as the finding revealed in the studies reported here (e) that DIET is feasible when only the electron-donating partner possesses e-pili. The results presented here do not support the concept that chains of magnetite (b) or cytochrome-to-cytochrome electron transfer (c) can support DIET.

Four basic mechanisms for DIET have been proposed (Fig. 1). Experimental evaluation for some of these proposed DIET strategies has been challenging due to a lack of appropriate microbial strains. A model for DIET in environments in which *Geobacter* species function as the electron-donating partner is that electrically conductive pili (e-pili [16]) are the conduit for long-range electron transport between cells (Fig. 1a). Although the e-pilus protein filaments are intrinsically conductive (16), multiheme cytochromes (17) or magnetite (18, 19) associated with the e-pili is important for DIET because it facilitates electron transfer between e-pili and donors or acceptors (20, 21). The e-pili DIET model is based on the finding that DIET-based consortia that involve *Geobacter* species form electrically conductive aggregates that contain dense e-pili networks (5, 6, 17, 22). Under physiologically relevant conditions, the conductivity of just one e-pilus (23, 24) is calculated to be sufficient to support half-maximal rates of extracellular electron transfer (25). Cells produce multiple e-pili. Thus, the density, conductivity, and physical flexibility of e-pili in DIET aggregates provide a high possibility that an electron-donating partner can make an electrical contact with an electron-accepting partner. Furthermore, DIET-based cocultures could not be established when the gene for PilA, the pilin monomer protein that assembles into e-pili in *Geobacter* species (26), was deleted (7, 17, 27, 28).

However, inhibiting e-pili expression by deleting *pilA* could eliminate e-pilus functions other than long-range electron transport. For example, the *Geobacter* pili may also play a role in establishing cell-to-cell contacts in biofilms (29). A better strategy for evaluating the importance of e-pili as an electrical contact for DIET is to construct *Geobacter* strains that produce poorly conductive pili (25, 30–32). The synthetic pilin gene *aro-5* encodes a pilin monomer in which alanine is substituted for five aromatic amino acids important for electron transport along the pilus (30, 33, 34). Substituting *aro-5* for *pilA* in Geobacter sulfurreducens yielded *G. sulfurreducens* strain Aro-5, which expressed abundant pili and properly localized its outer surface cytochromes (30). However, the conductivity of individual pili of strain Aro-5 (38 µS/cm at pH 7) was 3 orders of magnitude lower than the conductivity of wild-type pili (23). As the result of low pilus conductivity, *G. sulfurreducens* strain Aro-5 was incapable of long-range electron transport to Fe(III) oxides or the production of high current densities on electrodes (30).

*Geobacter* strains with poorly conductive pili could also provide experimental tools for examining proposed alternative mechanisms for DIET. For example, another suggested model for DIET (Fig. 1b) is that multiple magnetite particles can form conductive chains between *Geobacter* species and their DIET partners (35–39). However, convincing physical evidence for electrical connections through chains of magnetite is lacking (5), and studies with various *Geobacter* mutants suggested that the actual function of magnetite is to serve as a surrogate for pilus-associated *c*-type cytochromes that facilitate short-range electron transfer between e-pili and other electron acceptors or donors (18).

A third model for DIET (Fig. 1c) was proposed to describe interspecies electron transfer through anaerobic methanotrophic (ANME) consortia anaerobically oxidizing methane with the reduction of sulfate (12, 14). The genomes of both partners in the consortia encode multiple multiheme cytochromes, and abundant cytochromes were visualized on the outer cell surfaces (12, 14). Critical to this model is the assumption that cells can produce enough outer surface cytochromes to form a conductive matrix. However, as previously reviewed in detail (4), it has not been demonstrated that the aggregates are conductive or that outer surface/extracellular cytochromes are sufficiently abundant to form long-range cytochrome-to-cytochrome electrical contacts. Possibilities for experimental evaluation of the cytochrome-to-cytochrome hypothesis have been limited because the microbes within the ANME consortia have yet to be recovered in pure culture. However, the multiheme cytochromes proposed to enable cytochrome-to-cytochrome electron transfer within the ANME consortia have high homology to *Geobacter c*-type cytochromes (12, 14). Thus, *Geobacter* strains with poorly conductive pili and the ability to produce abundant outer surface *c*-type cytochromes offer an experimental model to evaluate whether microorganisms can adapt for DIET via cytochrome-to-cytochrome electron transfer.

In a fourth model (Fig. 1d), conductive carbon materials such as granular activated carbon (GAC) (40), biochar (41), or carbon cloth (42) facilitate long-range electron transfer by serving as an electrical conduit between the two DIET partners. e-pili are not required (28, 40, 42). Cells attach to the conductive materials, rather than to their DIET partners, suggesting that the cells “plug into” the conductive material (Fig. 1d). In *Geobacter*, these electrical contacts are presumably made with one or more of the *c*-type cytochromes that are abundant on the outer surface of *Geobacter* species (43, 44). Thus, DIET in the presence of GAC functions as a positive control to demonstrate that the strains being investigated retain the capacity for extracellular electron exchange.

Here, we evaluate the four previously proposed models for DIET with *Geobacter* strains with poorly conductive pili. The results demonstrate a fifth possibility (Fig. 1e) that electron-sharing partners can establish biological electrical contacts if only the electron-donating partner possesses e-pili. The results do not support the concepts of DIET via magnetite chains or cytochrome-to-cytochrome electron transfer without e-pili.

## RESULTS AND DISCUSSION

**FIG 2:**
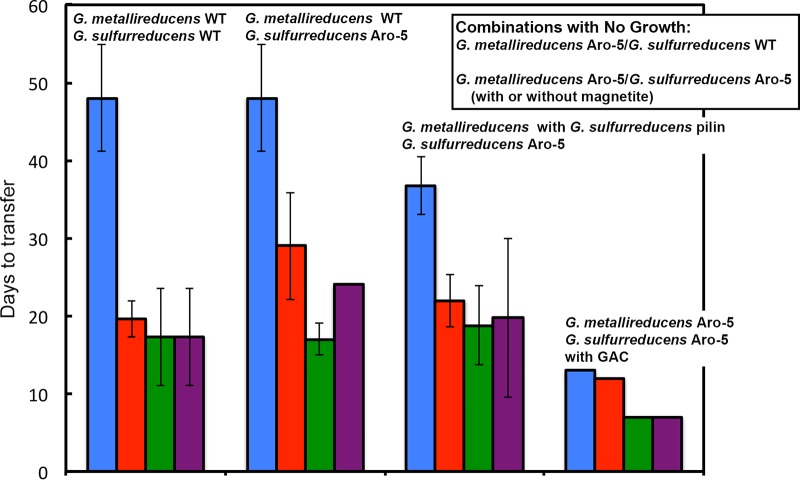
Potential for different strain combinations to grow via DIET. Time required for cocultures initiated with various strains of G. metallireducens and G. sulfurreducens to reduce ca. 20 mM fumarate to succinate at initiation of cocultures (first bar) and three successive transfers. Abbreviations: WT, wild type; GAC, granular activated carbon. The results are the means and standard deviations for triplicate cocultures. Error bars for GAC-amended cultures are too small to be visualized.

Studies were conducted with cocultures in which Geobacter metallireducens was the electron-donating partner and G. sulfurreducens was the electron-accepting partner. The cocultures were grown in medium with ethanol as the electron donor and fumarate as the electron acceptor. Coculture growth is possible only via DIET under these conditions because G. metallireducens can oxidize ethanol but cannot use fumarate as an electron acceptor and G. sulfurreducens can use fumarate as an electron acceptor but cannot metabolize ethanol (17). As previously reported (17), cocultures established with wild-type strains of both species initially required more than 30 days to initially adapt for effective DIET (Fig. 2). Once adapted, the DIET-based metabolism was faster in subsequent transfers (Fig. 2).

### The G. sulfurreducens strain with poorly conductive pili grows as the electron-accepting partner for DIET.

**FIG 3:**
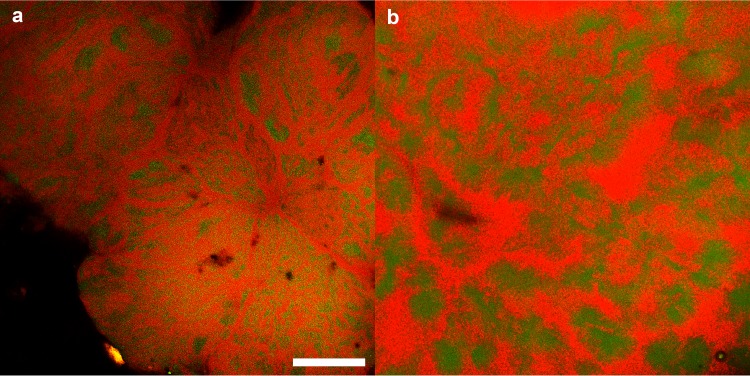
Confocal scanning laser micrograph of coculture aggregates. Cocultures initiated with wild-type G. metallireducens and either *G. sulfurreducens* strain Aro-5 (a) or wild-type G. sulfurreducens (b). The aggregates were treated with fluorescent *in situ* hybridization (FISH) probes specific for each species. G. metallireducens is shown in green, and G. sulfurreducens is shown in red. Bar, 50 µm.

Cocultures initiated with wild-type G. metallireducens and the Aro-5 strain of G. sulfurreducens grew as well as cocultures initiated with the wild type of both strains (Fig. 2). The coculture formed aggregates like those observed in cocultures with wild-type G. sulfurreducens, and cells were organized within the aggregates in a manner (Fig. 3) similar to that previously reported for aggregates formed with cocultures initiated with wild-type G. sulfurreducens and G. metallireducens (17).

The ability of the G. sulfurreducens Aro-5 strain to form DIET-based cocultures with G. metallireducens contrasts with the previously described (17) inability of a *pilA* deletion strain of G. sulfurreducens to form cocultures with G. metallireducens. These results suggest that G. sulfurreducens requires pili, even if they are poorly conductive, to aid in promoting contact with G. metallireducens and the formation of the coculture aggregates.

By the fourth transfer, it appeared that the coculture with the two wild-type strains might be metabolizing ethanol faster than the G. metallireducens*/*G. sulfurreducens Aro-5 coculture. To evaluate this further, the cocultures were continually transferred. After 11 successive transfers, the G. metallireducens*/*G. sulfurreducens Aro-5 coculture was metabolizing ethanol at 1.5 mM/day, whereas the contemporaneous culture with both wild-type strains had adapted to metabolize ethanol at 3.3 mM/day. This result suggested that, after long-term adaption, interspecies electrical connections can be enhanced when both DIET partners possess e-pili.

### G. metallireducens expressing the poorly conductive pili is limited in extracellular electron transfer.

**FIG 4:**
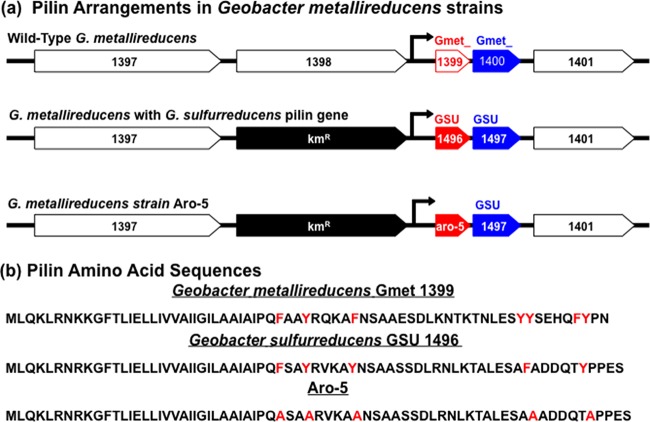
Gene organization surrounding pilin genes in G. metallireducens strains (a) and associated pilin amino acid sequences (b). Gene numbers are from the genomes of G. metallireducens (Gmet_1397, _1398, _1399, _1400, and _1401; NCBI reference sequence NC_007517.1) or G. sulfurreducens (GSU1496 and -1497; NCBI reference sequence NC_002939.5). Gmet_1399 and GSU1496 are the pilin genes (*pilA*). *aro-5* designates the synthetic pilin gene from G. sulfurreducens in which codons for five aromatic amino acid residues were replaced with those for alanine. Km^r^ is a kanamycin resistance gene. The arrows indicate the predicted locations of the promoter region for the pilin and associated downstream gene. Gmet_1398 encodes the transposase of ISGme6 in the ISL3 family. Red highlights aromatic amino acids considered to be important for electron transport along the pilus or where alanine was substituted for aromatic amino acids. The DNA sequences for the constructions are available in Fig. S1.

**FIG 5:**
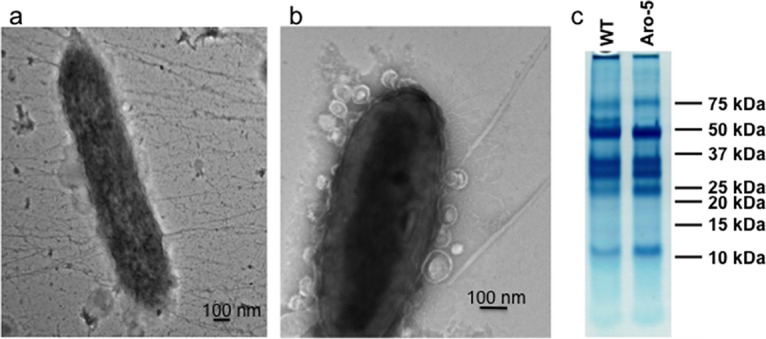
Pili and *c*-type cytochromes of G. metallireducens expressing the Aro-5 pilin gene. (a and b) Transmission electron micrographs of *G. metallireducens* strain Aro-5. (c) SDS-PAGE of loosely associated outer surface protein preparation of G. metallireducens wild-type strain (WT) and Aro-5 strain stained for *c*-type heme-containing proteins.

The finding that DIET was feasible with an electron-accepting partner lacking e-pili led to the question of whether an electron-donating partner without e-pili could participate in DIET. Therefore, an Aro-5 strain of G. metallireducens was constructed (Fig. 4). The wild-type pilin gene (Gmet_1399) and the adjacent downstream gene (Gmet_1400) of G. metallireducens were replaced with the synthetic *aro-5* gene and G. sulfurreducens gene GSU1497, which is located downstream of the wild-type G. sulfurreducens pilin gene (GSU 1496) in the G. sulfurreducens genome. *G. metallireducens* strain Aro-5 continued to produce pili (Fig. 5a and b) and expressed an array of abundant outer surface *c*-type cytochromes that were comparable to the wild type in molecular weight and abundance (Fig. 5c). One heme-staining band at ca. 58 kDa that was apparent in the wild type was fainter in the Aro-5 strain, but this band does not correspond to any of the *c*-type cytochromes found to be important in extracellular electron transfer in G. metallireducens (45).

**FIG 6:**
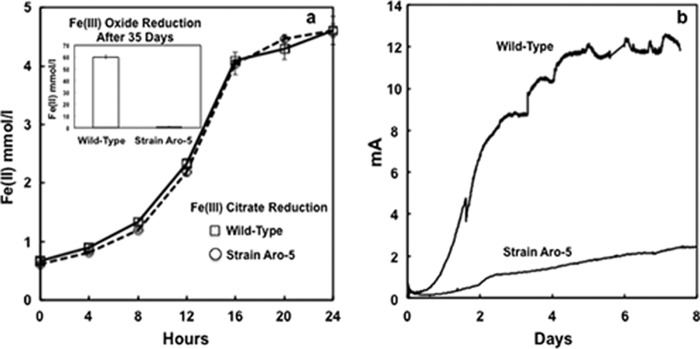
Fe(III) reduction and current production by *G. metallireducens* strain Aro-5 and the wild-type strain. (a) Production of Fe(II) over time from Fe(III)-citrate and Fe(II) produced from Fe(III) oxide after 35 days. Results are the means from triplicate cultures for the Fe(III) citrate cultures and quadruplicate cultures for the Fe(III) oxide cultures. (b) Representative current production by *G. metallireducens* strain Aro-5 and the wild-type strain.

*G. metallireducens* strain Aro-5 reduced Fe(III) citrate as well as wild-type G. metallireducens but was ineffective in Fe(III) oxide reduction (Fig. 6a). It produced much lower current densities than the wild type (Fig. 6b). These phenotypes are similar to *G. sulfurreducens* strain Aro-5 and are expected in a strain expressing pili with low conductivity (30). The results are consistent with the previous suggestion (45, 46) that G. metallireducens requires e-pili for long-range extracellular electron transfer.

### G. metallireducens requires e-pili to function as the electron-donating partner.

Cocultures could not be established with *G. metallireducens* strain Aro-5 and wild-type G. sulfurreducens. However, a strain of G. metallireducens in which *aro-5* was replaced with the wild-type *pilA* of G. sulfurreducens grew via DIET just as fast as cocultures initiated with wild-type G. metallireducens, even with *G. sulfurreducens* strain Aro-5 as the electron-accepting partner (Fig. 2). These results demonstrated that heterologous expression of pilin genes in G. metallireducens can yield a strain capable of participating in DIET when the pilin gene encodes a pilin that can assemble into e-pili and that G. metallireducens requires e-pili in order to effectively function as the electron-donating partner for DIET.

### Cytochromes or magnetite is not sufficient for DIET in the absence of e-pili.

Cocultures initiated with the Aro-5 strains of both G. metallireducens and G. sulfurreducens are good tests for the potential for DIET based on cytochrome-to-cytochrome electron transfer or magnetite-mediated DIET because both DIET partners lack e-pili but have abundant outer surface cytochromes to mediate extracellular electron exchange. Cocultures could not be established with the Aro-5 strains after multiple attempts with incubations lasting over a year. However, both of the Aro-5 strain partners had the ability for effective extracellular electron exchange as evidenced by rapid growth via DIET when the cultures were amended with GAC (Fig. 2).

Cocultures of the two Aro-5 strains could not be grown when magnetite was added. This result is consistent with the concept that magnetite does not form a conduit for long-range extracellular electron transfer but rather functions as a cytochrome surrogate to facilitate electron exchange with e-pili (5, 18).

### Implications.

The results do not support the hypotheses that either cytochrome-to-cytochrome electron transfer or electron transport through chains of magnetite can sustain DIET. The positive control with added GAC demonstrated that the Aro-5 strains of G. metallireducens and G. sulfurreducens possessed the necessary electrical contacts to facilitate rapid extracellular electron exchange. Yet, in the absence of GAC, the cocultures initiated with both Aro-5 strains did not adapt for DIET via their abundant outer surface cytochromes, even when magnetite was added. Experimental evidence for cytochrome-to-cytochrome or magnetite conduit DIET models has been lacking in previous studies (5), and thus, these models remain speculative.

It is probably physically challenging for two species to effectively position cytochromes on the cell surface close enough (<2 nm) to other extracellular cytochromes to enable DIET based on cytochrome-to-cytochrome electron transfer (4, 16). In contrast, dense networks of long, flexible e-pili have a high probability of establishing cell-to-cell electrical contacts, and just one e-pilus may be sufficiently conductive to satisfy the electron transfer needs of the cell (23–25). GAC overcomes the challenge of two cells achieving direct physical contact between outer surface cytochromes by providing a large, readily accessible conductive surface for independent cytochrome contact by both DIET partners. Soluble electron shuttles, such as quinones, are another strategy to overcome the physical difficulties in making direct electrical contacts between redox-active proteins fixed on the outer surface of cells (47), but extracellular release of soluble shuttles is unlikely to be adaptive in open environments due to diffusive loss of the shuttle (48). It might be possible for the electron-donating partner to “enclose” its partner, as has been suggested for some anaerobic methane-oxidizing consortia (15). In this way, shuttles could be maintained within the consortium and transport electrons between cytochromes analogously to quinone-based electron transport between electron transport proteins in microbial membranes.

There is not yet enough information to speculate why DIET was possible when only the electron-donating partner possessed e-pili but not when just the electron-accepting partner expressed e-pili. However, the finding that DIET is possible with electron-accepting partners that do not have e-pili suggests that it will not be surprising if the electrical contacts for DIET in *Methanothrix* (formerly *Methanosaeta*) (7) and *Methanosarcina* (28) species accepting electrons from G. metallireducens are not conductive filaments. In a similar manner, recent studies (32) suggested that the pili of a sulfate reducer proposed to “wire” a thermophilic ANME consortium for DIET (13) were poorly conductive, but DIET might still be a possibility if, as recently proposed (5, 15), the electron-donating partner expresses conductive filaments. These results demonstrate the need to further explore the diversity of e-pili in the microbial world.

## MATERIALS AND METHODS

### Strains and growth conditions.

Wild-type strains of G. metallireducens (49, 50) and G. sulfurreducens (51, 52) and *G. sulfurreducens* strain Aro-5 (30) were obtained from our laboratory collection. *G. metallireducens* strain Aro-5 and a G. metallireducens strain expressing the pilin monomer of wild-type G. sulfurreducens were constructed as described below. All *Geobacter* strains were grown under anaerobic conditions at 30°C in a defined medium with acetate as the electron donor and Fe(III) citrate, Fe(III) oxide, or fumarate as the electron acceptor, as previously described (50, 52). Escherichia coli DH5α (53) was used for plasmid preparation and grown in LB medium (54), supplemented with appropriate antibiotics, when necessary.

### Construction of *G*. *metallireducens* strain Aro-5 or with G. sulfurreducens PilA.

*G. metallireducens* strain Aro-5 was constructed by replacing Gmet_1399 and Gmet_1400 genes with the previously described (30) gene *aro-5* and GSU1497, the gene adjacent to the wild-type pilin monomer gene in G. sulfurreducens (Fig. 4a). *aro-5* encodes a pilin monomer in which five aromatic amino acids that play a key role in electron transfer along the assembled pili were replaced with alanine (Fig. 4b). The replacement was achieved with double-crossover homologous recombination (52). A DNA fragment of the upstream region for the double-crossover homologous recombination was amplified by PCR with the primer pair of 5′ TCTCTAGAAGGTCCTCGTCCGTAAC 3′ (XbaI site underlined) and 5′ TCTGAATTCATTCCTCATCCATCCCGAAC 3′ (EcoRI site underlined). The downstream region was amplified with the primer pair of 5′ TCTGTCGACGCTGTTCATGCTTGATAC 3′ (SalI site underlined) and 5′ TCTGGTACCGGCCGATTAGCCATCTG 3′ (KpnI site underlined). A DNA fragment of a kanamycin resistance gene was amplified by PCR with the primer pair of 5′ TCTGAATTCCTGACGGAACAGCGGGAAGTC 3′ (EcoRI site underlined) and 5′ TCTAAGCTTCATAGAAGGCGGCGGTGGAATC 3′ (HindIII site underlined) and pBBR1MCS-2 (55) as a template. DNA fragments (PpilA/aro-5 or GSU1496/GSU1497) containing a putative promoter region (PpilA) of the G. metallireducens pilin gene (Gmet_1399), the *aro-5* gene or GSU1496, and GSU1497 (see Fig. S1 in the supplemental material) were synthesized (Invitrogen). These DNA fragments were digested with restriction enzymes, ligated, and cloned in a plasmid. Plasmids thus constructed were linearized by XbaI. The linearized DNA fragments were used to replace Gmet_1399 and Gmet_1400 with *aro-5* or GSU1496 and GSU1497. The replacement by double-crossover homologous recombination in G. metallireducens was conducted as described previously (52) except that Fe(III) citrate was used instead of fumarate as the electron acceptor in the growth medium. Gmet_1398, located upstream of Gmet_1399, which encodes a transposase of ISGme6 in the ISL3 family, was replaced with the kanamycin resistance gene (Fig. 4a).

10.1128/mBio.01273-18.1 FIG S1  Sequence of PpilA/GSU1496 or aro-5/GSU1497. Coding regions are indicated in blue. Initiation and stop codons are indicated in bold. Codons for the five aromatic amino acid residues and their alanine substitutions are indicated in red. Putative ribosome binding sites are underlined. Highly conserved dinucleotides GG and GC in putative −24/−12 promoter elements for the RNA polymerase sigma factor RpoN are indicated in green. Predicted binding sites for PilR, a putative transcription factor in the RpoN-dependent enhancer-binding protein family, are indicated in green with underlines. AAGCTT and GTCGAC are recognition sequences for HindIII and SalI, respectively. Download FIG S1, DOCX file, 0.2 MB. Copyright © 2018 Ueki et al. 2018 Ueki et al. This content is distributed under the terms of the Creative Commons Attribution 4.0 International license.

The correct replacement was confirmed by cloning and sequencing the replaced region. A primer pair of 5′ AGTGCCTGCAAGTGGCGCTATATC 3′ and 5′ ACGGCACCAACATCATTCAC 3′ was used to amplify the region, and the amplified region was cloned in pCR-Blunt II-TOPO (Invitrogen) for Sanger sequencing.

### Cocultures.

Cocultures were grown in pressure tubes in 10 ml of medium in which ethanol (20 mM) was the electron donor and fumarate (40 mM) was the electron acceptor (17). Succinate in the cocultures was measured every 5 to 7 days, and a 1% inoculum was transferred into fresh medium if succinate production from fumarate reduction was ≥15 mM. GAC (8 to 20 mesh; 0.25 g) or magnetite (20 to 50 nm; 5 mmol liter^−1^) (18) was added to the culture medium when noted, at concentrations found previously (18, 40) to optimally promote DIET.

### Microscopy.

For transmission electron microscopy, cells were stained with uranyl acetate and examined as previously described (24). Cell aggregates were examined with fluorescent *in situ* hybridization (FISH) and confocal scanning laser microscopy as previously described (17) with the following modifications. (i) Ten percent formamide was used in the hybridization buffer. (ii) The aggregates were hybridized in 36 µl of hybridization buffer with 3 µl of each probe (G. sulfurreducens, 5′-[Cy5]GAAGACAGGAGGCCCGAAA-3′, and G. metallireducens, 5′-[Cy3]AGAATCCAAGGACTCCGT-3′ [17]) and 1 µl of each helper (G. sulfurreducens 5′-GTCCCCCCCTTTTCCCGCAAGA-3′ and 5′-CTAATGGTACGCGGACTCATCC-3′ [56]; G. metallireducens 5′-GAAGGTCCCCCCCTTTTCCCGC-3′ and 5′-GGGCTTATCCGGTATTAGCACC-3′ [57]). All probe and helper concentrations were 10 µM. (iii) Aggregates were hybridized in a 1.5-ml microcentrifuge tube. (iv) After washing, liquid was removed and aggregates were rinsed with 80% ethanol and deionized water and air dried on a slide, before mounting with a 4:1 ratio of CitiFluor to Vecta Shield (58). Aggregates were visualized with a Leica TCS SP5 microscope (Leica Microsystems GmbH, Wetzlar, Germany) with an HCX PL FLUOstar L 40× (numerical aperture 0.6) objective as previously described (17).

### Current production.

The capacity of the strains to produce current was determined as previously described (59). Cells were grown in two-chambered H-cell systems with a continuous flow of medium with acetate (10 mM) as the electron donor and graphite stick anodes (65 cm^2^) poised at 300 mV versus Ag/AgCl as the electron acceptor.

### Analytical techniques.

Succinate was measured with high-performance liquid chromatography as previously described (60). Fe(II) concentrations were determined by the ferrozine assay as previously described (49). *c*-type cytochromes associated with the outer membrane were determined as previously described (61).
