# Supplementary material for: Geobacter Strains Expressing Poorly Conductive Pili Reveal Constraints on Direct Interspecies Electron Transfer Mechanisms
Source: mBio. 2018 Jul 10;9(4):e01273-18. doi: 10.1128/mBio.01273-18 (PMC6050967; doi:10.1128/mBio.01273-18)
Supplement: FIG S1 [file mbo004183970sf1.docx]

>PpilA/GSU1496/GSU1497

aagctt

AGTGCCTGCAAG**TGGCGCTATATCACA**CACTTGGCGACTTGCCAGAACCCCTGTGTCACC

ACAGATTCTGCTAAAGAGCCTGATTTAGGAGAG**TGACAAATTTTGTCA**TGGCTGAGTGAC

CAAAGATGTGAAGTTTTTTTTTGTGTGAGTAAGCAGGTTGAAATTATTGGTGTTTTGGTG

TATGAATGATT**GG**CCTGGCTTTT**GC**TAAATGAGAAGCAACGTTTAAGGACTAAGCGGATA

ATTGGCCAAGTATCCTGATTCCAAGACGCACCAGAAGCACACACAGGAAAGGAGATGCAC

**ATG**CTTCAGAAACTCAGAAACAGGAAAGGTTTCACCCTTATCGAGCTGCTGATCGTCGTT

GCGATCATCGGTATTCTCGCTGCAATTGCGATTCCGCAG**TTC**TCGGCG**TAT**CGTGTCAAG

GCG**TAC**AACAGCGCGGCGTCAAGCGACTTGAGAAACCTGAAGACTGCTCTTGAGTCCGCA

**TTT**GCTGATGATCAAACC**TAT**CCGCCCGAAAGT**TAA**

TGCAAGAAGTTTCTGTTGTCTCTGATAACCGCTCGAGACGAAAGGAGTTATTT

**ATG**AAAAAGATCATTACTATAGTTGCTATGTTGCTCGCAATGCAGGGAATAGCCATTGCC

GCCGGGAAAATTCCTACAACAACGATGGGTGGCAAGGACTTTACTTTCAAACCTTCTACT

AACGTGAGTGTTTCCTACTTCACTACGAACGGTGCAACTTCTACCGCGGGAACTGTCAAT

ACCGATTATGCTGTCAATACCAAAAACTCTTCTGGTAACCGGGTGTTCACCTCAACCAAT

AATACATCTAACATCTGGTACATCGAAAATGATGCATGGAAAGGTAAGGCAGTTTCAGAT

AGCGATGTTACCGCCTTGGGAACCGGTGACGTAGGCAAGTCTGATTTTTCTGGTACCGAG

TGGAAGTCGCAG**TAG**

gtcgac

>PpilA/aro-5/GSU1497

aagctt

AGTGCCTGCAAG**TGGCGCTATATCACA**CACTTGGCGACTTGCCAGAACCCCTGTGTCACC

ACAGATTCTGCTAAAGAGCCTGATTTAGGAGAG**TGACAAATTTTGTCA**TGGCTGAGTGAC

CAAAGATGTGAAGTTTTTTTTTGTGTGAGTAAGCAGGTTGAAATTATTGGTGTTTTGGTG

TATGAATGATT**GG**CCTGGCTTTT**GC**TAAATGAGAAGCAACGTTTAAGGACTAAGCGGATA

ATTGGCCAAGTATCCTGATTCCAAGACGCACCAGAAGCACACACAGGAAAGGAGATGCAC

**ATG**CTTCAGAAACTCAGAAACAGGAAAGGTTTCACCCTTATCGAGCTGCTGATCGTCGTT

GCGATCATCGGTATTCTCGCTGCAATTGCGATTCCGCAG**gcC**TCGGCG**gcT**CGTGTCAAG

GCG**gcC**AACAGCGCGGCGTCAAGCGACTTGAGAAACCTGAAGACTGCTCTTGAGTCCGCA

**gcT**GCTGATGATCAAACC**gcT**CCGCCCGAAAGT**TAA**

TGCAAGAAGTTTCTGTTGTCTCTGATAACCGCTCGAGACGAAAGGAGTTATTT

**ATG**AAAAAGATCATTACTATAGTTGCTATGTTGCTCGCAATGCAGGGAATAGCCATTGCC

GCCGGGAAAATTCCTACAACAACGATGGGTGGCAAGGACTTTACTTTCAAACCTTCTACT

AACGTGAGTGTTTCCTACTTCACTACGAACGGTGCAACTTCTACCGCGGGAACTGTCAAT

ACCGATTATGCTGTCAATACCAAAAACTCTTCTGGTAACCGGGTGTTCACCTCAACCAAT

AATACATCTAACATCTGGTACATCGAAAATGATGCATGGAAAGGTAAGGCAGTTTCAGAT

AGCGATGTTACCGCCTTGGGAACCGGTGACGTAGGCAAGTCTGATTTTTCTGGTACCGAG

TGGAAGTCGCAG**TAG**

gtcgac

**Supplementary Fig. 1. Sequence of PpilA/GSU1496 or aro-5/GSU1497**

Coding regions are indicated in blue. Initiation and stop codons are indicated in bold. Codons for the five aromatic amino acid residues and their alanine substitutions are indicated in red. Putative ribosome binding sites are underlined. Highly conserved dinucleotides GG and GC in putative -24/-12 promoter elements for the RNA polymerase sigma factor RpoN are indicated in green. Predicted binding sites for PilR, a putative transcription factor in the RpoN-dependent enhancer-binding protein family, are indicated in green with underlines (Krushkal, J., Juárez, K., Barbe, J. F., Qu, Y., Andrade, A., Puljic, M., Adkins, R. M., Lovley, D. R., and **Ueki, T**. 2010. Genome-wide survey for PilR recognition sites of the metal-reducing prokaryote *Geobacter sulfurreducens*. *Gene*. 469: 31-44.). aagctt and gtcgac are recognition sequences for HindIII and SalI, respectively.
